# Sagittal and pelvic arameters analysis in patients with adolescent idiopathic scoliosis

**DOI:** 10.1186/1748-7161-7-S1-O15

**Published:** 2012-01-27

**Authors:** S Donzelli, F Zaina, S Negrini

**Affiliations:** 1 ISICO, Milan, Italy

## Background and purpose

The sagittal alignment of the spine and pelvis in adolescent idiopathic scoliosis is poorly defined in the literature [1-8]. The purpose of this study was to assess the sagittal alignment in scoliosis patients according to curve degree and type.

## Material and methods

Sagittal parameters of the spine and pelvis were analysed in lateral standing radiographs of 256 adolescents (13.7±5 years, curve range 4-57) and compared with statistically normal values (NV) in adolescents found in the literature: thoracic kyphosis TK (NV 22–66), lumbar lordosis LL (NV 24-72), pelvic incidence PI (NV 27-71), sacral slope SS (NV 25-57) and pelvic tilt PT (NV –8-16). Lateral standing radiographs were matched with anteroposterior radiograph. Patients were classified according to the entity of scoliosis curves, age, gender and risser score.

## Results

There is a weak negative correlation (0.2) between scoliosis and kyphosis. Over 20° Cobb PI increased, mainly due to an increase of the SS. In our population we had low PI and SS, but mainly in less than 20° curves than in higher scoliosis; on the contrary, PT was high in all children. Analysing curves type and decrease of SS we found that this occurs more frequently in patients with double curves (thoracic and thoracolumbar).

## Conclusions

PI increases through life, and curves degree worsen with growth, and this influence our results. Patients with spinal deformities have a positive sagittal balance and signs of pelvic retroversion such as decreased SS. According to our data this situation occurs to patients with thoracic and thoracolumbar curves.
